# EDENT1FI Master Protocol for screening of presymptomatic early-stage type 1 diabetes in children and adolescents

**DOI:** 10.1136/bmjopen-2024-088522

**Published:** 2025-01-02

**Authors:** Luisa Hoffmann, Mirjam Kohls, Stefanie Arnolds, Peter Achenbach, Regine Bergholdt, Ezio Bonifacio, Emanuele Bosi, Melanie Gündert, Bianca K Hoefelschweiger, Sandra Hummel, Przemysława Jarosz-Chobot, Olga Kordonouri, Vito Lampasona, Parth Narendran, Lut Overbergh, Flemming Pociot, João Filipe Raposo, Zdeněk Šumník, Agnieszka Szypowska, Jurgen Vercauteren, Christiane Winkler, Chantal Mathieu, Anette-Gabriele Ziegler, the EDENT1FI consortium, C Mathieu

**Affiliations:** 1 Institute of Diabetes Research, Helmholtz Munich German Research Center for Environmental Health, Munich, Germany; 2 Forschergruppe Diabetes at Klinikum Rechts der Isar, Technical University of Munich School of Medicine, Munich, Germany; 3 Novo Nordisk A/S, Soeborg, Denmark; 4 Center for Regenerative Therapies Dresden, Faculty of Medicine, TU Dresden, Dresden, Germany; 5 Diabetes Research Institute, IRCCS Ospedale San Raffaele, Milan, Italy; 6 Vita-Salute San Raffaele University, Milan, Italy; 7 Department of Children's Diabetology, Faculty of Medical Sciences in Katowice, Medical University of Silesia, Katowice, Poland; 8 Kinderkrankenhaus auf der Bult, Hanover, Germany; 9 University of Birmingham, Birmingham, UK; 10 Department of Chronic Diseases and Metabolism, KU Leuven, Leuven, Belgium; 11 Department of Clinical Research, Translational Type 1 Diabetes Research, Steno Diabetes Center Copenhagen, Herlev, Denmark; 12 NOVA Medical School, Faculdade de Ciências Médicas, Universidade Nova de Lisboa, Lisbon, Portugal; 13 Education and Research Center (APDP-ERC), APDP-Diabetes Portugal, Lisbon, Portugal; 14 Department of Pediatrics, Second Faculty of Medicine, Charles University in Prague and Motol University Hospital, Prague, Czech Republic; 15 Department of Pediatrics, Medical University of Warsaw, Warsaw, Poland; 16 Department of Endocrinology, University Hospitals Leuven, Leuven, Belgium

**Keywords:** General diabetes, Paediatric endocrinology, PUBLIC HEALTH, Community child health

## Abstract

**Abstract:**

**Introduction:**

The identification of type 1 diabetes at an early presymptomatic stage has clinical benefits. These include a reduced risk of diabetic ketoacidosis (DKA) at the clinical manifestation of the disease and a significant reduction in clinical symptoms. The European action for the Diagnosis of Early Non-clinical Type 1 diabetes For disease Interception (EDENT1FI) represents a pioneering effort to advance early detection of type 1 diabetes through public health screening. With the EDENT1FI Master Protocol, the project aims to harmonise and standardise screening for early-stage type 1 diabetes and care.

**Methods and analysis:**

Public health islet autoantibody screening is conducted in the Czech Republic, Denmark, Germany, Italy, Poland, Portugal, Sweden and the UK. Between November 2023 (start date) and October 2028 (planned end date), an estimated number of 200 000 children and adolescents aged 1–17 years are expected to be screened. Screening is performed in capillary blood, examining different islet autoantibodies (autoantibodies against insulin, glutamic acid decarboxylase-65, insulinoma-associated antigen-2 and/or zinc transporter-8). Positive screening results undergo confirmation through a second antibody method. A second (venous) blood sample is requested if at least two autoantibodies are detected, to confirm the autoantibody status. Children and adolescents with confirmed two or more autoantibodies are invited to metabolic staging (oral glucose tolerance test, haemoglobin A1c (HbA1c), random glucose, optionally continuous glucose monitoring); an educational programme and recommendations for monitoring are provided. The feasibility and acceptability of screening are evaluated by feedback questionnaires. Pseudonymised data is collated in the EDENT1FI Registry. Study outcomes include country-specific screening rates, prevalences of stage 1 and stage 2 type 1 diabetes, number in EDENT1FI Registry, proportion with DKA and symptoms at clinical diagnosis and median HbA1c.

**Ethics and dissemination:**

Following the EDENT1FI Master Protocol, site-specific protocols are developed and approved by local ethics committees (Technical University of Munich, Medical Faculty, Nr. 70/14; Medizinische Hochschule Hannover, Nr. 9588_BO_S_2021; Technische Universität Dresden, Nr. BO-EK-356082020; Center for Sundhed Region Hovedstaden, Nr. H-22053116; Swedish Ethical Review Authority, Nr. 2023-00312-01; National Health Service Health Research Authority and Health Care Research Wales, IRAS (Integrated Research Application System) project ID 309252; Italian National Institute of Health, National ethics committee for clinical trials of public research bodies (EPR) and other national public institutions, Prot. PRE BIO CE Nr. 0059835; Charles University in Prague, Ethics Committee for Multi-Centric Clinical Trials of the University Hopital Motol and 2nd Faculty of Medicine, Nr. 1271/23; Bioethics Committee at the Medical University of Warsaw, Nr. 21/2024 and KB/6/R/2024; Associação Protectora dos Diabéticos de Portugal, Nr. 211/2024). Results are disseminated through peer-reviewed journals and conference presentations and will be shared openly.

**STRENGTHS AND LIMITATIONS OF THIS STUDY**

- Template and recommendation for public health screening for islet autoantibodies.
- Quality-controlled test strategy with confirmation of a positive screening result by two tests in two consecutive samples.
- Application in eight European countries with different healthcare settings.
- Assessment of feasibility and acceptability of screening in families and healthcare providers.
- Restricted data on multiple ethnic populations; current lack of a companion therapy for prevention.

## Introduction

 Type 1 diabetes is the most common metabolic disorder in children and imposes a lifelong burden on patients and their caregivers. The incidence and severity of type 1 diabetes have been escalating over recent decades, a trend that has been particularly noticeable since the onset of the COVID-19 pandemic. Importantly, type 1 diabetes is associated with significant morbidity and an elevated mortality risk, with children developing type 1 diabetes before the age of 10 years losing on average 16 years of life expectancy.^1 2^

At diagnosis, many patients, especially children, experience substantial morbidity. This often necessitates hospitalisation and admission to intensive care units due to a common complication, diabetic ketoacidosis (DKA). Despite numerous educational efforts, DKA affects up to 50% of children at the initial presentation of clinical type 1 diabetes (www.sweet-project.org).^3^ DKA is not only a life-threatening condition at the time of diagnosis, but emerging evidence demonstrates a lifelong impact on metabolic control and cognitive function.^4^,^9^ Furthermore, insulin replacement therapy, the standard treatment for type 1 diabetes, often fails to achieve physiological glycaemic control despite significant advancements in insulin analogues, glucose monitoring and insulin delivery technologies.

As a consequence of these learnings and as suggested in the recent guidelines from the International Society for Pediatric and Adolescent Diabetes,^10^ a century after the first clinical use of insulin, there is an urgent need to change the paradigm of diagnosis and therapy of type 1 diabetes. Key priorities include early detection of type 1 diabetes (ie, when the autoimmune disease is already present, as evidenced by circulating autoantibodies to multiple islet antigens but before the onset of hyperglycaemia) through population screening; and in tandem, to advance the development and appropriate use of disease-modifying therapies as a direct means of reducing type 1 diabetes and its associated morbidity, mortality and overall burden to individuals and society.

### Islet autoantibodies as robust biomarkers for the diagnosis of early-stage type 1 diabetes

The detection of islet autoantibodies is widely acknowledged as a robust, sensitive and specific biomarker for the diagnosis of early-stage type 1 diabetes.^11 12^ The international consensus holds that the presence of two or more islet autoantibodies in the absence of hyperglycaemia represents the presymptomatic early stage of type 1 diabetes^10 13 14^ and European Medicines Agency has given a qualification opinion on the use of two or more islet autoantibodies as enrichment biomarkers for selecting patients in clinical trials investigating therapies to prevent or delay the clinical diagnosis of type 1 diabetes (https://www.ema.europa.eu/en/documents/regulatory-procedural-guideline/qualification-opinion-islet-autoantibodies-aas-enrichment-biomarkers-type-1-diabetes-t1d-prevention-clinical-trials_en.pdf). The specificity and sensitivity of islet autoantibodies as a biomarker depends on a testing strategy that examines three or four different types of islet autoantibodies (autoantibodies against insulin (IAA), glutamic acid decarboxylase-65 (GADA), insulinoma-associated antigen-2 (IA-2A) and/or zinc transporter 8 (ZnT8-A). This strategy employs two different methods and requires two blood samples taken at different times to confirm positive results.^15^,^17^ Most individuals with type 1 diabetes develop islet autoantibodies in childhood. Screening once for the presence of multiple islet autoantibodies has the highest sensitivity at age 4 years; this detects 40% of all cases of type 1 diabetes by age 15 years. Screening twice (eg, at ages 2 and 6) results in a sensitivity of>80%.^18 19^ The positive predictive value (PPV) of testing positive for multiple islet autoantibodies is close to 100%, that is, almost all children with a positive screening result develop clinical type 1 diabetes within 20 years. The PPV for clinical type 1 diabetes within 10 years is 75% and 79% by age 15 years.^11^ Reversion of two or more islet autoantibodies is extremely rare and was less than 1% in the TEDDY (The Environmental Determinants of Diabetes in the Young) study.^20^

### Screening for islet autoantibodies has clinical benefits for families

The Fr1da study in Bavaria^1517 21^,^26^ pioneered public health screening for early-stage type 1 diabetes in childhood and demonstrated that diagnosing type 1 diabetes at an early stage has clinical benefits for families.^23 27 28^ At the clinical onset of the disease, these include, as also found in previous studies, a drastically reduced risk of DKA at clinical manifestation of the disease;^2327^,^35^ a significant reduction in clinical symptoms and no weight loss at clinical manifestation of the disease;^27^ an improved beta cell function (C-peptide) and remarkedly lower frequency of metabolic abnormalities (haemoglobin A1c (HbA1c), glucose) at disease manifestation.^36^,^40^ An additional benefit is access to disease-modifying therapies such as teplizumab (Tzield^®^) which may become available in Europe in the near future and is approved in the USA.^41^,^46^

### Objectives

Following the Fr1da study as a model, the overall objective of European action for the Diagnosis of Early Non-clinical Type 1 diabetes For disease Interception (EDENT1FI) is to improve clinical care for children and adolescents with type 1 diabetes by diagnosing the disease at an early presymptomatic stage. The EDENT1FI Master Protocol addresses three components: (1) public health screening for islet autoantibodies to identify children and adolescents with early-stage type 1 diabetes; (2) staging of children and adolescents diagnosed with early-stage type 1 diabetes by oral glucose tolerance test, HbA1c, random glucose, optionally also continuous glucose monitoring; and (3) for families of children and adolescents with early-stage type 1 diabetes, an educational programme and recommendation for monitoring and follow-up. It will serve as a template for implementation in participating countries with some variation in age and setting. The EDENT1FI consortium is developing protocols addressing the assessment of psychological stress during screening and monitoring, longitudinal follow-up and long-term monitoring and intervention therapies. Specific objectives and outcomes are outlined in table 1.

**Table 1:** Objectives and outcomes of the EDENT1FI-screening Master Protocol

Objectives	Outcomes
Establish a network of primary care physicians/paediatricians who participate in the study and perform the screening.	Number of participating primary care physicians/paediatricians.
Inform the network of physicians and the public at large about the purpose of the study.	Dissemination material.
Screen up to 200 000 children and adolescents at ages ranging between 1 and 17 years between 2024 and 2028 and determine the prevalence of early-stage type 1 diabetes in predefined age groups.	Number of screened children and adolescents per month and year and region.Number of people with early-stage type 1 diabetes in EDENT1FI Registry per month and year and region.
Perform education and staging and enrol into monitoring protocol (separate consent) in children and adolescents with early-stage type 1 diabetes and their families.	Number of people with early-stage type 1 diabetes who participate in education and staging.Enrolment rate into tandem protocols (monitoring, assessment of psychological stress, interventions).
Evaluate the feasibility and acceptability of screening by feedback questionnaires.	Feasibility and acceptability of screening.
Register children and adolescents with early-stage type 1 diabetes (stage 1 or stage 2) into the EDENT1FI Registry.	Number of people in the registry.
Improve clinical outcomes (DKA, HbA1c, symptoms of hyperglycaemia, weight loss, days of hospitalisation, beta cell function) in children and adolescents with early-stage type 1 diabetes.*	Proportion of children and adolescents with DKA at clinical diagnosis.*Median HbA1c at clinical diagnosis (stage 3).*Per cent of children and adolescents with symptoms at clinical diagnosis (stage 3).*Median kg weight loss at clinical diagnosis (stage 3).*Days of hospitalisation at clinical diagnosis (including days at ICU).*Random or fasting C-peptide during oral glucose tolerance test at clinical diagnosis.*Insulin requirement at clinical diagnosis stabilising hyperglycaemia.*
Assess prior participation in newborn screening through GPPAD (if applicable).	Proportion of prior participation in newborn screening for type 1 diabetes risk (if applicable).
Assess parallel screening outcomes for other autoimmune diseases (frequency of coeliac-associated and/or thyroid-associated autoantibodies; if applicable).	Number of children and adolescents with coeliac-autoimmunity and/or thyroid-autoimmunity (if applicable).
Evaluate novel point of care testing for islet autoantibodies (if available).	Data on point of care testing (if available).
Adapt staging and follow-up guidelines as appropriate from findings within EDENT1FI and international screening studies.	

* wWill be assessed in context with other tandem protocols.

DKA, diabetic ketoacidosis; EDENT1FIEuropean action for the Diagnosis of Early Non-clinical Type 1 diabetes For disease InterceptionGPPAD, Global Platform for the Prevention of Autoimmune DiabetesHbA1c, haemoglobin A1cICUintensive care unit

## Methods

The study is a cross-sectional analysis with a longitudinal follow-up for children and adolescents diagnosed with early-stage type 1 diabetes.

### Study population and setting

Public health screening for islet autoantibodies is being conducted in the Czech Republic, Denmark, Germany, Italy, Poland, Portugal, Sweden and the UK. Between November 2023 and October 2028, an estimated 200 000 children and adolescents at ages ranging between 1 and 17 years are expected to be screened. The figure was derived from estimates of the individual countries which was considered feasible. The screening will be performed by primary care physicians and paediatricians or paediatric hospitals, during preventive medical check-ups, other paediatric visits, hospital stays and school or home tests. The EDENT1FI Master Protocol will serve as a template; individual country-specific protocols will be developed and adapted to local healthcare settings. Major variations will be in the selected age range for screening, the healthcare provider (HCP) setting and the combination with other autoimmune diseases (table 2). In addition to the criteria defined in table 2, inclusion criteria for participation are residence in the respective screening regions and written informed consent signed by the custodial parent(s). The exclusion criterion is a prior diagnosis of diabetes mellitus. The first participant screened was on 1 November 2023. The last patient screened is planned for October 2028.

**Table 2:** Overview of screening sites and setting

Country	Region	Planned screening number	Screening age	Place of screening	Participation per child	Combination with other diseases	Local project name
**Czechia**	Nationwide	20 000	2–18 years	Primary care, paediatricians, home testing	Once	No	βetty
**Denmark/Sweden**	Oresund Skåne	40 000	Sweden:6–9 and 13–14 yearsDenmark:1–18 years*	Home testing	Once	Coeliac disease and autoimmune thyroid disease	DiaUnion
**Germany**	Bavaria, Saxony,Lower Saxony and Hamburg	60 000	2–10 years	Primary care, paediatricians, hospitals	Twice	No	Fr1da
**Italy**	Lombardia, Marche, Campania, Sardinia	20 000	1–17 years, focus on age 2, 6 and 10 years	Primary care, paediatricians	Once	Coeliac disease	D1ce
**Poland**	Area Warsaw and Katowice	30 000	2–17 years	Primary care, paediatricians, university hospitals	Once	No	EDENT1FI
**Portugal**	Area Lisbon	10 000	6–12 years	Primary school testing, community settings	Once	No	EDENT1FI
**UK**	Nationwide	20 000	3–13 years	Home testing, school testing, medical practices, community settings	Once	No	ELSA†

* Screening of children and adolescents with first-degree relatives with type 1 diabetes.

† Sample collection, testing and assessment of acceptability in ELSA differs from the methodology outlined in this EDENT1FI Master Protocol.

EDENT1FIEuropean action for the Diagnosis of Early Non-clinical Type 1 diabetes For disease InterceptionELSAEarLy Surveillance for Autoimmune diabetes

### Healthcare professional engagement

Blood sample collection for islet autoantibody determination and consent procedures will be carried out by either the local coordinating centre (EDENT1FI partner) or by HCPs (physicians and paediatricians) participating in this project. To facilitate enrolment in the study, information events are organised and HCPs are informed about the possibility of participating in a public health research project on screening for islet autoantibodies for early diagnosis of type 1 diabetes. This information will be disseminated via personal invitation letters, meetings, quality circles, congresses and social media. The purpose, benefits and possible harm of screening are explained. HCPs who participate in the study must confirm their participation in writing and declare that they agree to obtain consent from study participants or legal representatives, keep the consent in the participant’s records, collect capillary blood and in rare circumstances venous blood from the study participant, collect some sociodemographic information on the study participant and ship the samples to the local coordination centre/screening laboratory. The HCP may also inform the family about the screening result. To support engagement for the study, healthcare professionals and policymakers may be involved or asked to take on a patronage or ambassadorial role for the screening. Such engagement should help to develop strategies for how screening for islet autoantibodies can be incorporated into existing policies and healthcare systems. In addition, the public at large will be informed about the purpose of the study through campaigns, posters, websites and social media.

### Enrolment, blood sample collection and islet autoantibody measurement

**Figure 1:**
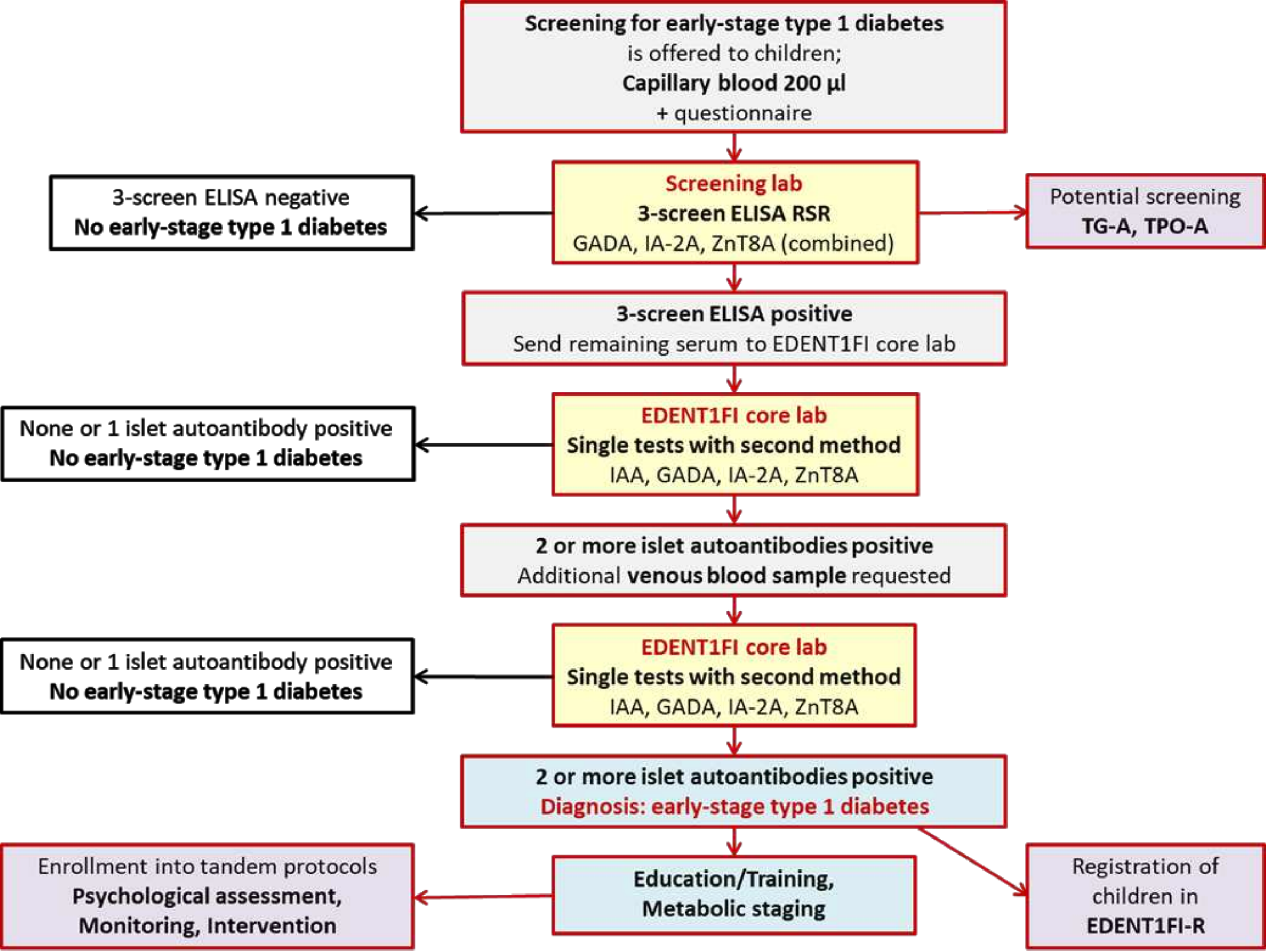
Flowchart showing the screening process for early-stage type 1 diabetes in EDENT1FI. Children and adolescents who have multiple islet autoantibodies detected in two different blood samples and by two different methods are diagnosed with early-stage type 1 diabetes. These individuals will be enrolled in the EDENT1FI Registry and invited to participate in an educational programme and metabolic staging. They will also be offered participation in tandem protocols examining the psychological burden of screening, conducting monitoring studies and intervention studies. EDENT1FI, European action for the Diagnosis of Early Non-clinical Type 1 diabetes For disease Interception; GADA, glutamic acid decarboxylase-65; IAA, autoantibodies against insulin; IA-2A, insulinoma-associated antigen-2; TG-A, transglutaminase autoantibodies; TPO-A, thyroid peroxidase autoantibodies; ZnT8A, zinc transporter 8.

The coordination centre/screening laboratory will provide tubes for capillary blood samples, consent forms, information material, posters and possibly incentives to the primary HCP (primary care physician/paediatrician) who performs the screening (figure 1). Written consent for study participation is obtained and remains with the HCP. A capillary blood sample of approximately 200 µl is taken (eg, finger berry, earlobe). Furthermore, a short questionnaire is filled out together with the parents. These materials are sent by post to the coordination centre/screening laboratory.

In the screening laboratory, the blood samples are processed and the serum is tested using a validated, sensitive first-line screening test (CE certified RSR 3-Screen ELISA^16 17^). The 98.5th percentile of values obtained in healthy children is used as a cut-off defining a positive test signal. For these study participants, residual blood or in case of low volume newly requested blood samples will be tested for IAA, GADA, IA-2A and ZnT8A using a second assay format like radiobinding assay or luciferase immunoprecipitation system assay or electrochemiluminescence assay. This confirmation testing is done by EDENT1FI core laboratories (Helmholtz Munich or San Raffaele Hospital (OSR) Milan). In case of two or more positive islet autoantibodies, an additional venous blood sample is requested for confirmation. Confirmation is again done by EDENT1FI core laboratories using the secondary assay format. Shipments to the core laboratories will be biweekly. If two or more positive islet autoantibodies are also present in the second blood sample, the diagnosis of early-stage type 1 diabetes is made. Additionally, coeliac disease-associated transglutaminase autoantibodies (TGA) and thyroid-peroxidase autoantibodies (TPO) may be tested locally.

There are international standards for autoantibodies and international workshops evaluating assay performance. Quality assessments of screening laboratories will be regularly performed by the EDENT1FI central laboratory teams. The EDENT1FI core laboratories are evaluated in world-standardisation workshops.^47^,^49^

The HCP who took the blood sample will be informed of the diagnosis and the number of positive islet autoantibodies. Results from the local assessment by 3-Screen ELISA and from the core laboratory will be entered into a central EDENT1FI database at Helmholtz Munich, Germany.

### Definition of early-stage type 1 diabetes

Early-stage type 1 diabetes is defined as being positive for at least two islet autoantibodies, confirmed positive by two different assay formats in two consecutive blood samples.

### Questionnaire

Sociodemographic data (age, sex, height, weight, first-degree family history status of type 1 diabetes) will be collected from all participants and entered in the EDENT1FI database.

### Staging, education and follow-up of children and adolescents diagnosed with early-stage type 1 diabetes

All children and adolescents diagnosed with early-stage type 1 diabetes and their parents will be asked to participate in an education and staging visit at the coordinating centre or any other collaborating paediatric diabetes care facilities. Collaborations with paediatric diabetes care facilities will be established. During the visit, an oral glucose tolerance test and/or HbA1c are performed to determine the disease stage and measure beta cell function (C-peptide). Depending on the result of these tests, a distinction is made between stage 1, stage 2 and stage 3 type 1 diabetes (online supplemental file 1)^13 14^ and therapy and minimal monitoring/follow-up are recommended accordingly (table 3).^50^

**Table 3:** Advice for minimal follow-up of individuals with early-stage type 1 diabetes after baseline oral glucose tolerance test and HbA1c for staging

Stage	Home glucose monitoring*	HbA1c monitoring	Islet autoantibody monitoring
**1 (or no staging**)	If symptoms only	6 monthly	6 monthly as long as IA-2A negative
**2**	1–3 monthly and if symptoms or infections	3 monthly	–

See also more detailed follow-up schemes described.^50^

* 1–2 hours post-carbohydrate rich meal.

HbA1chaemoglobin A1cIA-2Ainsulinoma-associated antigen-2

Individuals with the diagnosis of early-stage type 1 diabetes and their parents will be invited to participate in an educational training programme. The education takes place near the family’s home, if necessary, as a small group training with 2–3 other affected families. The training is carried out by a physician or qualified staff trained by EDENT1FI. Education will cover topics such as pathogenesis of type 1 diabetes, early-stage type 1 diabetes, symptoms of diabetes, blood glucose and urine glucose home monitoring, normal and abnormal glucose values, basic information and mode of action of insulin therapy, information on how to prevent possible mental stress, instruction on individual monitoring plan based on predicted disease progression and be appropriate for underserved communities. Families will be informed about disease-modifying therapies that are approved in the USA for the treatment of early-stage type 1 diabetes like teplizumab (Tzield^®^) or clinical trials aiming to test the efficacy of novel disease-modifying therapies and individuals will be invited to participate in such trials. Execution of such trials can happen in the context of INNODIA (www.innodia.org) or centres associated with the EDENT1FI project.

The families will be provided with a blood glucose meter or glucose sensor, a blood glucose diary or urine glucose test strips and will receive a monitoring plan with recommendations on the frequency of minimal metabolic and islet autoantibody control (HbA1c, glucose). In general, diabetes teams will be best equipped to monitor individuals with any stage of type 1 diabetes and we strongly recommend that individuals with early stage are followed in specialist care. However, individuals and their families may not wish to engage with specialist care until insulin therapy is indicated and may prefer monitoring by their primary care physician. Families will also receive a guidebook on early stages of type 1 diabetes (.typ1diabetes-frueherkennung.de/fileadmin/FRIEDA/PDF/Flyer_Poster_Formulare/Infos_in_Fremdsprachen/HH_Fr1da_InfoBrochure_EN_02.pdf).

The families are assigned a contact person (doctor or nurse) who they can contact in case of any questions or emergencies. If an individual develops stage 3 type 1 diabetes after a diagnosis of presymptomatic early-stage or after screening islet autoantibody negative, clinical parameters are recorded using a questionnaire (table 4).

**Table 4:** Clinical parameters captured in the questionnaire for type 1 diabetes onset*

Parameter	Values
**Symptoms**	Polyuria, polydipsia, weight loss in kg, infections, DKA.
**HbA1c and blood glucose values**	Fasting, postprandial or 2-hour value oral glucose tolerance test.
**Laboratory values of blood gas analysis**	pH, total CO2, pCO2, base deficit, base excess, potassium, sodium, chloride, BUN, plasma creatinine, beta-hydroxybutyrate.
**Beta cell function**	C-peptide random or fasting or during oral glucose tolerance test.
**Urine ketones**	Yes/no.
**Hospitalisation**	Outpatient or inpatient, number of days, days at ICU.
**Date of first insulin treatment**	Date.

* Not all variables have to be necessarily available.

BUNblood urea nitrogenCO2carbon dioxideDKA, diabetic ketoacidosisHbA1chaemoglobin A1cICUintensive care unitpCO2partial pressure of carbon dioxide

An assessment of psychological morbidity (HADS - Hospital Anxiety and Depression Scale) and impact on lived life (SDQ - Strengths and Difficulties Questionnaire) will be undertaken via previously validated questionnaires to identify those families that require further psychological support. A standardised risk assessment decision tree process will be implemented across all centres for families identified at risk to ensure timely and effective support is offered to those with possible psychological morbidity. This process will involve the assessment of PH-Q9 (Patient Health Questionnaire) or HADS with intervention provided to those scoring highly.

Healthcare professionals will be encouraged to report the development of stage 3 type 1 diabetes in children and adolescents who tested islet autoantibody negative.

Affected families are also offered participation in tandem protocols evaluating psychological stress of screening by questionnaire and qualitative interviews, performing monitoring studies and intercept prevention studies (figure 1). These tandem studies will require separate consent. As for newborn screening, negative islet autoantibody results are not necessarily communicated to the families. Children who have one single islet autoantibody will not be informed about a positive test result as the risk of progressing to clinical diabetes is low.

### Feasibility and acceptability of screening by feedback questionnaires

The feasibility and acceptability of screening for type 1 diabetes are assessed through various questionnaires suitable either for families or also for primary care HCP or paediatric diabetes care facilities (online supplemental file 2). These questionnaires will be made available as an online tool. The results may be used to improve study operations and communications throughout the project.

### EDENT1FI Registry for people with early-stage type 1 diabetes

The recently established Pre-T1D-REGISTRY (www.pre-t1d-registry.eu) collects data from people with early-stage type 1 diabetes across Europe in a pseudonymised manner. The registry is located and managed at Helmholtz Munich, Germany and already contains data from over 1000 persons with early-stage type 1 diabetes from INNODIA, the Global Platform for the Prevention of Autoimmune Diabetes (GPPAD), and Fr1da/Fr1da-Plex (Germany). The data include autoantibody and metabolic data, information on family history with type 1 diabetes, sex and age, and if available HLA-DR-DQ genotype.

### Prior participation in newborn screening through GPPAD (if applicable)

GPPAD is a primary prevention platform to identify infants with an elevated genetic risk of developing type 1 diabetes and to perform primary prevention trials aiming to reduce the incidence of islet autoimmunity and type 1 diabetes in children (www.gppad.org). Some individuals may have participated in newborn screening and/or primary prevention trials coordinated by GPPAD. In order to take advantage of prior information about the genetic risk of developing type 1 diabetes and allow integrated analyses of data obtained in GPPAD and EDENT1FI, we will ask families whether the child participated in GPPAD studies and whether data from GPPAD could be linked to data obtained here.

### Screening outcomes for other autoimmune diseases (frequency of coeliac-associated and/or thyroid-associated autoantibodies; if applicable)

In addition to the assessment of islet autoantibodies, some regions may also offer families to be tested for coeliac disease-associated TGA and for autoantibodies to thyroid peroxidase with additions to this protocol (TPO-A).

### Novel point-of-care testing for islet autoantibodies (if available)

Recently, lateral flow assays for islet autoantibodies in capillary blood and saliva have been developed. Accurate point-of-care tests would greatly improve logistics, reduce costs and accelerate diagnosis. Pilot activities that assess the sensitivity, specificity and accuracy of these tests are currently performed in the Munich central autoantibody laboratory and may be distributed to other screening sites for further evaluation. These tests would be applied in addition to the above-described testing procedures if remaining blood or sera is available.

### Statistics

The statistical analysis is conducted with established statistical programmes such as SAS (SAS Institute, Cary, North Carolina, USA), R (http://cran.r-project.org/) and SPSS. The outcomes as described above will be determined by the frequency and descriptive statistics, logistic regression or life table analyses.

### Risk and benefit evaluation

#### Benefits of diagnosing early-stage type 1 diabetes

At the time of type 1 diabetes diagnosis, many individuals, especially children, experience substantial morbidity, frequently requiring hospitalisation and intensive care unit admission due to severe metabolic derailment and DKA which affects up to half of children at first presentation of clinical type 1 diabetes.^3^ DKA is not only a life-threatening condition at the time of diagnosis, but emerging evidence demonstrates a lifelong impact on metabolic control and cognitive function.^4^,^9^ Despite many educational initiatives, DKA remains prevalent at clinical diagnosis of type 1 diabetes in 2024.

As summarised above for the Fr1da study and as also demonstrated in TEDDY and DAISY (Diabetes Autoimmunity Study in the Young), screening for early-stage type 1 diabetes reduces DKA incidence at the onset of clinical type 1 diabetes and thus reduces morbidity and mortality and will improve lives by reducing disease burden. Other patient-relevant benefits include the reduction of symptoms and weight loss, hospitalisation and improved beta cell function at clinical onset. Many families are currently overwhelmed by the diagnosis of symptomatic type 1 diabetes at stage 3, as they must deal simultaneously with blood glucose control, insulin dose adjustment, hypoglycaemia, acceptance of chronic disease in their child and understanding the disease. Early diagnosis at a presymptomatic stage allows for early medical treatment, targeted training, education, psychological preparation and gradual learning of insulin therapy and metabolic control. The development of hyperglycaemia is usually a slow process. Early diagnosis therefore leads to a significantly lower acute burden overall and reduces the risks of metabolic derailment. Prevention of symptomatic hyperglycaemia in type 1 diabetes is the most important goal of current research efforts. Early diagnosis of type 1 diabetes allows participation in intervention therapies to prevent clinical manifestation.

#### Risks of diagnosing early-stage type 1 diabetes

A possible risk of screening is the psychological stress in the case of a positive finding due to the knowledge of a chronic disease in the child. If this burden precedes the clinical manifestation of the disease for many years and affects a family over a prolonged period of years, this can possibly lead to disadvantages that counteract the shown benefits. Data from the BABYDIAB, TEDDY and Fr1da studies show that notification of a positive islet autoantibody result was associated with increased parental anxiety and worry which decreased over time to levels of the general population,^22 51 52^ confirming the findings of previous studies.^53 54^ The educational programme is designed to provide all families with a sense of security, a feeling that they are being looked after and that they are being cared for by experts.

The physical risks from participation are limited to those arising from capillary/venous blood sampling (pain on puncture, haematoma, infection, etc). Study participants are given the opportunity to minimise pain during puncture by applying an anaesthetic ointment beforehand.

### Data protection

The screening blood samples and the collected data will be pseudonymised. The data collected from the questionnaire will be entered or transferred into a central EDENT1FI database at Helmholtz Munich and made available for statistical analyses.

The study will comply with the General Data Protection Regulation and Data Protection Act 2018. The processing of the personal data of participants will be minimised by using a unique participant study number on all study documents and any electronic database(s). All documents will be stored securely and only accessible by study staff and authorised personnel. The study staff will safeguard the privacy of participants’ personal data.

### Ethics and dissemination

The study is conducted in accordance with relevant regulations and with Good Clinical Practice as applicable. Before study initiation, the country-specific or region-specific protocol and the informed consent documents have been reviewed and approved by the competent Ethics Committee (Technical University of Munich, Medical Faculty, Nr. 70/14; Medizinische Hochschule Hannover, Nr. 9588_BO_S_2021; Technische Universität Dresden, Nr. BO-EK-356082020; Center for Sundhed Region Hovedstaden, Nr. H-22053116; Swedish Ethical Review Authority, Nr. 2023-00312-01; National Health Service Health Research Authority and Health Care Research Wales, IRAS (Integrated Research Application System) project ID 309252; Italian National Institute of Health, National ethics committee for clinical trials of public research bodies (EPR) and other national public institutions, Prot. PRE BIO CE Nr. 0059835; Charles University in Prague, Ethics Committee for Multi-Centric Clinical Trials of the University Hopital Motol and 2nd Faculty of Medicine, Nr. 1271/23; Bioethics Committee at the Medical University of Warsaw, Nr. 21/2024 and KB/6/R/2024; Associação Protectora dos Diabéticos de Portugal, Nr. 211/2024). The protocol and informed consent have also been reviewed by the Chair of the Ethical Advisory Board of EDENT1FI. Results are disseminated through peer-reviewed journals and conference presentations and will be shared openly.

Families will be invited to participate in the study by a HCP or by the study team. Families will be given enough time to consider whether to participate in the study. Information material will be developed to inform or assent children and adolescents.

## supplementary material

10.1136/bmjopen-2024-088522 online supplemental file 1

10.1136/bmjopen-2024-088522 online supplemental file 2
