# Supplementary material for: EDENT1FI Master Protocol for screening of presymptomatic early-stage type 1 diabetes in children and adolescents
Source: BMJ Open. 2025 Jan 2;15(1):e088522. doi: 10.1136/bmjopen-2024-088522 (PMC11749223; doi:10.1136/bmjopen-2024-088522)
Supplement: online supplemental file 1 [file bmjopen-15-1-s001.pdf]

SUPPLEMENTARY FILE 1: CRITERIA FOR NORMOGLYCAEMIA, DYSGLYCAEMIA AND HYPERGLYCAEMIA

	**Normoglycaemia (stage 1)**	**Dysglycaemia (stage 2) ***	**Hyperglycaemia (stage 3) ****
**Fasting plasma glucose**	FPG <100 mg/dL (<5.6 mmol/l)	FPG 100-125 mg/dL (5.6-6.9 mmol/l)	FPG ≥126 mg/dL (≥7.0 mmol/l)
	OR	OR	OR
**Haemoglobin A1c**	HbA1c <5.7% (<39 mmol/mol)	HbA1c 5.7-6.4% (39-47 mmol/mol) OR<br>HbA1c ≥10% increase from previous visit	HbA1c ≥6.5% (≥48 mmol/mol)
	OR	OR	OR
**Oral glucose tolerance test**	2hr PG <140 mg/dL (<7.8 mmol/l)	2hr PG 140-199 mg/dL (7.8-11.0 mmol/l) OR<br>30-, 60- or 90-min PG ≥200 mg/dL (≥11.1 mmol/l)	2hr PG ≥200 mg/dL (≥11.1 mmol/l)
			OR
**Random plasma glucose**			Symptoms + PG ≥200 mg/dL (≥11.1 mmol/l)

* Stage 2 definition in EDENT1FI requires that 2 criteria are met at the same visit OR at least 1 criterion is met at two consecutive visits. ** In the absence of unequivocal hyperglycaemia, diagnosis requires 2 abnormal test results from the same sample or in two separate test samples. Abbreviations: FPG: fasting plasma glucose, HbA1c: haemoglobin A1c, PG: plasma glucose. For classification and staging of type-1-diabetes, see also [13, 14]
