# Supplementary material for: EDENT1FI Master Protocol for screening of presymptomatic early-stage type 1 diabetes in children and adolescents
Source: BMJ Open. 2025 Jan 2;15(1):e088522. doi: 10.1136/bmjopen-2024-088522 (PMC11749223; doi:10.1136/bmjopen-2024-088522)
Supplement: online supplemental file 2 [file bmjopen-15-1-s002.pdf]

SUPPLEMENTARY FILE 2: EVALUATION OF FEASIBILITY AND ACCEPTABILITY OF SCREENING FOR TYPE 1 DIABETES

Questionnaire 1: Feedback questionnaire for primary care paediatricians

Based on your experience with the [study name] study in recent years,...

1. How useful do you think the screening is from a medical point of view?

□ Very useful □ Somewhat useful □ Average □ Not particularly useful □ Not at all useful

2. How useful do you think the screening is for the families (including the opportunities and burden involved)?

□ Very useful □ Somewhat useful □ Average □ Not particularly useful □ Not at all useful

3. How useful would it be to integrate the screening permanently into the standard check-up program?

□ Very useful □ Somewhat useful □ Average □ Not particularly useful □ Not at all useful

4. How can the screening be integrated into the routine paediatric check-ups in your medical office?

□ Very good □ Good □ Satisfactory □ Poor □ Very poor

5. How would you rate the information you have received from the coordinating centre about the Fr1da study?

□ Very good □ Good □ Satisfactory □ Poor □ Very poor

6. How would you rate the availability of the coordinating centre to answer questions?

□ Very good □ Good □ Satisfactory □ Poor □ Very poor

7. How would you rate the service provided by the coordinating centre (materials, responses provided)?

□ Very good □ Good □ Satisfactory □ Poor □ Very poor

8. Have one or more children in your practice been diagnosed with an early stage of type 1 diabetes?

□ No □ Yes (please also answer the following questions*)

*9. How would you rate the information on diagnosis provided by the coordinating centre?

□ Very good □ Good □ Satisfactory □ Poor □ Very poor

*10. How would you rate the referral of the family to a paediatric diabetes care centre for training and consultation?

□ Very good □ Good □ Satisfactory □ Poor □ Very poor

*11. How would you rate the support provided by the coordinating centre for the ongoing care of the family and the child?

□ Very good □ Good □ Satisfactory □ Poor □ Very poor

*12. How would you rate the current support provided by the paediatric diabetes care centre to the family and the child?

□ Very good □ Good □ Satisfactory □ Poor □ Very poor

Questionnaire 2: Feedback questionnaire for paediatric diabetes care centres

Based on previous experience of caring for children with diagnosis of type 1 diabetes at pre-symptomatic early stage compared to symptomatic stage in recent years,...

1. How would you assess the burden on families dealing with the diagnosis?

□ Significantly less □ Somewhat less □ Similar □ Somewhat greater □ Much greater

2. How would you assess the amount of work you have to do to provide the initial training?

□ Significantly less □ Somewhat less □ Similar □ Somewhat more □ Much more

3. How would you estimate the amount of work you have to do to provide the initial treatment?

□ Significantly less □ Somewhat less □ Similar □ Somewhat more □ Much more

4. How would you estimate the amount of work you have to do to provide long-term care?

□ Significantly less □ Somewhat less □ Similar □ Somewhat more □ Much more

5. How would you rate the training materials for the early-stage type 1 diabetes?

□ Very good □ Good □ Satisfactory □ Poor □ Very poor

6. How would you rate working together with the coordinating centre (materials, responses provided)?

□ Very good □ Good □ Satisfactory □ Poor □ Very poor

7. How would you rate the availability of the coordinating centre to answer questions?

□ Very good □ Good □ Satisfactory □ Poor □ Very poor

8. How would you rate the information you have received from the coordinating centre about the [study name] study?

□ Very good □ Good □ Satisfactory □ Poor □ Very poor

9. How would you rate the current support provided by the coordinating centre for children with an early stage of type 1 diabetes?

□ Very good □ Good □ Satisfactory □ Poor □ Very poor

10. How useful do you think [study name] screening is from a medical point of view?

□ Very useful □ Somewhat useful □ Average □ Not particularly useful □ Not at all useful

Questionnaire 3: Feedback questionnaire for parents 6 months after educational training

How satisfied are you with your decision to participate in the [study name] study?

1. How satisfied are you in general that your child is currently participating in the Fr1da study?

□ very satisfied □ satisfied □ partly □ unsatisfied □ very unsatisfied

2. Do you think that it was a good decision to participate in the [study name] study?

□ very good decision □ good decision □ okay □ bad decision □ very bad decision

3. Would you recommend the [study name] study to other parents with young children?

□ definitely yes □ generally yes □ depends □ not really □ no, definitely not

How satisfied are you with the support provided by the primary care paediatricians, paediatric diabetes care centres and the [study name] coordinating centre?

How satisfied are you with…

1. …the information provided about the study prior to your participation in the study?

□ very satisfied □ satisfied □ partly □ unsatisfied □ very unsatisfied

2. … the communication of the diagnosis “early stage type 1 diabetes”?

□ very satisfied □ satisfied □ partly □ unsatisfied □ very unsatisfied

3. … the first educational training about “pre-symptomatic type 1 diabetes” you received after diagnosis?

□ very satisfied □ satisfied □ partly □ unsatisfied □ very unsatisfied

4. … the possibility to reach somebody to answer your questions?

□ very satisfied □ satisfied □ partly □ unsatisfied □ very unsatisfied

5. … the organization of the [study name] study?

□ very satisfied □ satisfied □ partly □ unsatisfied □ very unsatisfied

6. … the written information provided?

□ very satisfied □ satisfied □ partly □ unsatisfied □ very unsatisfied

Questionnaire 4: Feedback questionnaire for parents 12 months after educational training

How satisfied are you with your decision to participate in the [study name] study?

1. How satisfied are you in general that your child is currently participating in the [study name] study?

□ very satisfied □ satisfied □ partly □ unsatisfied □ very unsatisfied

2. Do you think that it was a good decision to participate in the [study name] study?

□ very good decision □ good decision □ okay □ bad decision □ very bad decision

3. Would you recommend the [study name] study to other parents with young children?

□ definitely yes □ generally yes □ depends □ not really □ no, definitely not

How satisfied are you with the support provided by the primary care paediatricians, paediatric diabetes care centres and the [study name] coordinating centre?

How satisfied are you with…

1. … the possibility to reach somebody to answer your questions?

□ very satisfied □ satisfied □ partly □ unsatisfied □ very unsatisfied

2. … the overall support provided by the [study name] study team?

□ very satisfied □ satisfied □ partly □ unsatisfied □ very unsatisfied
