# Supplementary material for: EDENT1FI Master Protocol for screening of presymptomatic early-stage type 1 diabetes in children and adolescents
Source: BMJ Open. 2025 Jan 2;15(1):e088522. doi: 10.1136/bmjopen-2024-088522 (PMC11749223; doi:10.1136/bmjopen-2024-088522)
Supplement: Supplementary file 3 [file bmjopen-2024-088522.reviewer_comments.pdf]

PEER REVIEW HISTORY

BMJ Open publishes all reviews undertaken for accepted manuscripts. Reviewers are asked to complete a checklist review form and are provided with free text boxes to elaborate on their assessment. These free text comments are reproduced below.

ARTICLE DETAILS

Title (Provisional)

The EDENT1FI Master Protocol for Screening of Presymptomatic Early-Stage Type 1 Diabetes in Children and Adolescents

Authors

Hoffmann, Luisa; Kohls, Mirjam; Arnolds, Stefanie; Achenbach, Peter; Bergholdt, Regine; Bonifacio, Ezio; Bosi, Emanuele; Gündert, Melanie; Hoefelschweiger, Bianca K; Hummel, Sandra; Jarosz-Chobot, Przemysława; Kordonouri, Olga; Lampasona, Vito; Narendran, P; Overbergh, Lut; Pociot, Flemming; Raposo, João Filipe; Šumník, Zdeněk; Szypowska, Agnieszka; Vercauteren , Jurgen; Winkler, Christiane; Mathieu, Chantal; Ziegler, Anette-Gabriele; consortium, EDENT1FI

VERSION 1 - REVIEW

**Reviewer**	**1**
**Name**	**Hess Fischl, Amy**
**Affiliation**	**University of Chicago, Endocrinology**
**Date**	**14-Aug-2024**
**COI**	**na**

The authors commented that positive antibodies may have psychological impact on the subjects. Since this is a global issue, it is recommended that the authors include some commentary regarding next step education plans for these possibilities.

**Reviewer**	**2**
**Name**	**Joshi, Kriti**
**Affiliation**	**Queensland Children's Hospital**
**Date**	**03-Nov-2024**
**COI**	**None**

Reviewer comments

Manuscript ID: bmjopen-2024-088522

The EDENT1FI Master Protocol for Screening of Presymptomatic Early-Stage Type 1 Diabetes in Children and Adolescents

General comments:

Well thought out and comprehensively reviewed protocol with a clear framework, plan for implementation and well specified goals of screening for T1D risk.

Certain important aspectes of methodology need further clarification as below

Methods:

1. Age at screening different in all regions, so that will impact on prevalence data. Are the authors planning on calculating country specific prevalence?
2. Table 2: How is the planned screening number calculated? Is it based on population size or previous prevalence data from the region.
3. Exclusion criteria to also include current participation in an intervention trial for pre-symptomatic diabetes, use of any immunomodulatory/ immunosuppressive treatments.
4. List of health care partners/coordinating centres. Are these primary health care providers or specialists?
5. ?Consider Involvement of GP practices, immunisation clinics, maternal and child health nurses to disseminate information.
6. Will all screening be performed in the primary practice by the HCP involved or are family's given the option of doing the DBS testing at home and posting it to the coordinating lab? Some families might want to take time to consider and review trial information before consenting to study participation and therefore gaining their consent at the first point of contact may not be feasible. Will home based testing be an option?
7. Consider QR code based consenting procedures.
8. IAA not being included in the initial screen might reduce the sensitivity of the test in picking up younger individuals at risk of T1D
9. Protocol for offering re-screening to individuals negative on first testing is unclear. Given the wide age ranges it is plausible that individuals negative on first screen might subsequently develop islet auto-antibodies. What will be done to address this? Alternatively, is a standard age of screening recommended?
10. Single autoantibody positive individuals particularly those with IAA+ or high titre of other AAs. Will there be any follow up provided to this cohort? Should ideally be brought in line with international consensus (Phillip et all 2024).
11. TPO and Coeliac testing protocol needs to be elaborated further. At what stage is this offered, at the first step or only to those who screen positive for islet autoantibodies
12. Staging and follow up: should be brought in line with the latest international consensus, Phillip et al 2024. < 3 yrs should have metabolic monitoring every 3 months
13. Details of OGTT? Feasibility of doing this in the younger children and consideration for alternative methods of glycaemic monitoring i.e. CGM?
14. What are the funding provisions for ongoing monitoring and glucose meters, metabolic testing/ clinic visits as this will vary from region to region.
15. What guidelines/safety netting will be provided to families regarding development of Stage 3 and seeking medical intervention. Stage 3a can be a challenging stage and timing of insulin initiation is controversial so when is specialist intervention recommended?
16. Table 4: will an oral glucose tolerance test be done for all patients even with Stage 3b T1D? Do the authors mean a MMTT in the context of a Stage 3 intervention trial?

17. Details of guidebook and psychological support provided to families? Is there a separate protocol for this?
18. Details of Data management software used

VERSION 1 - AUTHOR RESPONSE

The authors commented that positive antibodies may have psychological impact on the

Reviewer: 1
Dr. Amy Hess Fischl, University of Chicago Comments to the Author:
subjects. Since this is a global issue, it is recommended that the authors include some commentary regarding next step education plans for these possibilities.
A short paragraph has been added to explain how psychological impact and anxiety is assessed and what action is taken. However as outlined, there will be additional projects to investigate this by qualitative interview and questionnaires that use a separate consent and protocol.

Reviewer: 2
Dr. Kriti Joshi, Queensland Children's Hospital

We thank the reviewer for the constructive suggestions toward a protocol. We would like to point out that this is a composite protocol that has been approved by ethical review boards in each of the participating countries. Indeed, the manuscript did not go out for review until the journal was satisfied that ethical approval had been obtained from all participating sites. We are, therefore, unable to modify the study protocol. We have, however, responded and where appropriate modified the manuscript as described below.

Comments to the Author:

1. Age at screening different in all regions, so that will impact on prevalence data. Are the authors planning on calculating country specific prevalence? *Yes. As already outlined in the objectives, we will look at “prevalence of early- stage type 1 diabetes in pre-defined age groups”. We now added that numbers per region will also be assessed.*

2. Table 2: How is the planned screening number calculated? Is it based on population size or previous prevalence data from the region.
It is calculated on estimates of what was considered feasible within this time period. This has been added.

3. Exclusion criteria to also include current participation in an intervention trial for presymptomatic diabetes, use of any immunomodulatory/ immunosuppressive treatments.
This manuscript is on a protocol that has been approved by ethics boards and is in use. The content of the protocol itself cannot be changed. We do not exclude people under immunomodulatory treatment for other diseases as these may have islet autoantibodies.

4. List of health care partners/coordinating centres. Are these primary health care providers or specialists?
EDENT1FI partners are specialists. As stated, depending on the countryspecific setting, blood collection will be done by both:
“Blood sample collection for islet autoantibody determination, and consent procedures will be carried out by either the local coordinating centre (EDENT1FI partner) or by health care providers (physicians and
paediatricians) participating in this project″

5. ?Consider Involvement of GP practices, immunisation clinics, maternal and child health nurses to disseminate information.
Thank you for these suggestions. As said, the protocol is not for review, and it was requested by the journal that ethical approval is already obtained before submission.
6. Will all screening be performed in the primary practice by the HCP involved or are family’s given the option of doing the DBS testing at home and posting it to the coordinating lab?
This varies and is provided in table 2, column place of screening.

Some families might want to take time to consider and review trial information before consenting to study participation and therefore gaining their consent at the first point of contact may not be feasible. Will home based testing be an option?
Only in specific regions, see Table 2

7. Consider QR code based consenting procedures.
Not in line with regulations in most Eu countries; as stated above, the protocol is not to be changed.

8. IAA not being included in the initial screen might reduce the sensitivity of the test in picking up younger individuals at risk of T1D
This is not correct as we are screening for early-stage type 1 diabetes, not for single insulin autoantibodies.

9. Protocol for offering re-screening to individuals negative on first testing is unclear. Given the wide age ranges it is plausible that individuals negative on first screen might subsequently develop islet auto-antibodies. What will be done to address this? Alternatively, is a standard age of screening recommended?

This protocol was developed before the consensus was published, and it is mostly in line with the consensus. Many authors of this manuscript are authors of the consensus paper. The

As outlined in table 2, there is no standard age. It is left to the regional decision what is most feasible. This protocol is for rolling out screening and elaborating acceptance and feasibility in various settings. It is for one screen and funded for one screen.

10. Single autoantibody positive individuals particularly those with IAA+ or high titre of other AAs. Will there be any follow up provided to this cohort? Should ideally be brought in line with international consensus (Phillip et all 2024).
There is no follow-up offered for people with single autoantibodies as these autoantibodies are highly unspecific and often transient. This protocol is a research protocol with different questions that a consensus document for regular care. This is a research study and not regular care.

11. TPO and Coeliac testing protocol needs to be elaborated further. At what stage is this offered, at the first step or only to those who screen positive for islet autoantibodies
The EDENT1FI core protocol is only about screening for islet autoantibodies. It is left to the individual region decision to combine it with other screenings and therefore it is mentioned but not elaborated because it is not part of the core protocol. This has now been clarified.

12. Staging and follow up: should be brought in line with the latest international consensus, Phillip et al 2024. < 3 yrs should have metabolic monitoring every 3 months
protocol cannot be changed in the middle of the project as this will cause inconsistencies. This is a research study and not regular care.

13. Details of OGTT? Feasibility of doing this in the younger children and consideration for alternative methods of glycaemic monitoring i.e. CGM? *Longitudinal studies and cross sectional screening studies including TEDDY, DIPP, GPPAD, TrialNet have all demonstrated that OGTT is feasible also in small children. If an OGTT cannot be performed, staging will be done by HbA1c.This has now been clarified.*
As outlined, there will be a separate protocol for monitoring including CGM; this protocol includes an initial OGTT for staging and a minimal monitoring for families who decide not to consent to the monitoring protocol.

14. What are the funding provisions for ongoing monitoring and glucose meters, metabolic testing/ clinic visits as this will vary from region to region. *Monitoring is funded by the IHI grant. The costs per site are based on a standard cost estimate.*

15. What guidelines/safety netting will be provided to families regarding development of Stage 3 and seeking medical intervention. Stage 3a can be a challenging stage and timing of insulin initiation is controversial so when is specialist intervention recommended?
Specialist intervention is recommended for all early stage T1D care. This has been clearly stated.

16. Table 4: will an oral glucose tolerance test be done for all patients even with Stage 3b T1D? Do the authors mean a MMTT in the context of a Stage 3 intervention trial?
Table 4 variables are captured if available, but not all need to be necessarily assessed. This has been added.

17. Details of guidebook and psychological support provided to families? Is there a separate protocol for this?

Yes, there is a separate protocol not part of this manuscript. A link to a guidebook is now provided and the names of the standardized psychological questionnaires provided.

18. Details of Data management software use
We use an adapted software solution from Qubicon (https://qubicon.io/).

We look forward to your response.
